# Natural disasters and indicators of social cohesion

**DOI:** 10.1371/journal.pone.0176885

**Published:** 2017-06-07

**Authors:** Aitor Calo-Blanco, Jaromír Kovářík, Friederike Mengel, José Gabriel Romero

**Affiliations:** 1 Dpto. de Economía, Universidade da Coruña, Campus de A Coruña, 15071 A Coruña, Spain; 2 Dpto. Fundamentos Análisis Económico I & Bridge, Universidad del País Vasco/Euskal Herriko Unibersitatea, Av. Lehendakari Aguirre 83, 48015 Bilbao, Spain; 3 CERGE-EI, a joint workplace of Charles University Prague and the Economics Institute of the Czech Academy of Sciences, Politických vězňů 7, 111 21 Prague, Czech Republic; 4 Department of Economics, University of Essex, Wivenhoe Park, Colchester CO43SQ, United Kingdom; 5 Departamento de Economía, Universidad de Santiago de Chile, Alameda 3363, Santiago, Chile; Queensland University of Technology, AUSTRALIA

## Abstract

Do adversarial environmental conditions create social cohesion? We provide new answers to this question by exploiting spatial and temporal variation in exposure to earthquakes across Chile. Using a variety of methods and controlling for a number of socio-economic variables, we find that exposure to earthquakes has a positive effect on several indicators of social cohesion. Social cohesion increases after a big earthquake and slowly erodes in periods where environmental conditions are less adverse. Our results contribute to the current debate on whether and how environmental conditions shape formal and informal institutions.

## Introduction

There is widespread agreement that social cohesion can be crucial to a society’s cultural and economic development. Social cohesion lowers the transaction costs of working together and hence facilitates cooperation. People have the confidence to invest in collective activities, knowing that others will also do so [1–3]. They are also less likely to engage in private actions with negative outcomes for society as a whole [4–6]. While the importance of social cohesion is widely acknowledged, there is little agreement on why some countries or regions have seemingly higher levels of social cohesion than others [7–9].

Proponents of “environmentalism” believe that environmental conditions are key determinants in shaping human formal and informal institutions (e.g. [10]) and thus argue that adversarial environmental conditions create social cohesion [8, 9, 11, 12]. The idea is that harsher environmental conditions lead people to be more cooperative either because they understand the necessity of cooperating in order to survive, or because stronger evolutionary pressures select for more cooperative communities [8, 13–15]. Harsher environmental conditions, however, could also lead to increased competition for scarce resources [16], more conflict and as a result less social cohesion [17–19]. Understanding which effect prevails is difficult, because it is hard to find geographical areas with largely identical formal institutions, but substantial exogenous variation in environmental conditions.

**Fig 1:**
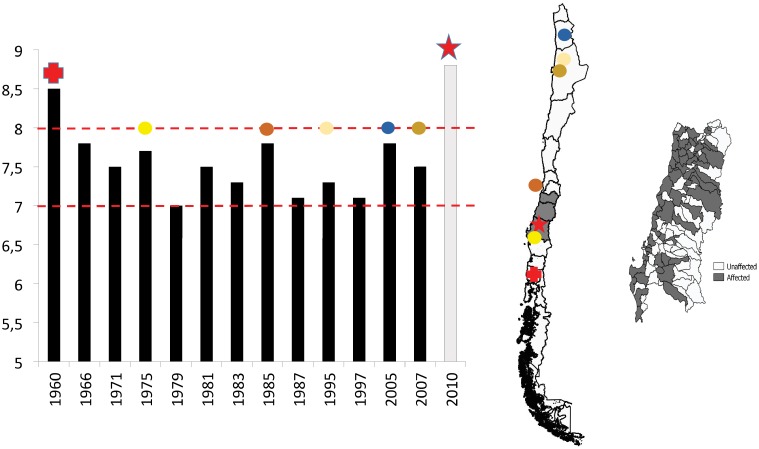
Earthquakes in Chile. Chile is administratively divided into 15 regions and 346 *comunas*. Left: temporal distribution of some major earthquakes in Chile between 1960–2010, measured in *Ms* (except for the 2010 Maule event for which only *Mw* is available). Center: a map of Chile with approximate epicenters of some of these earthquakes. Right: cross-*comuna* variation in exposure to the 2010 Maule earthquake (*Mw*8.8) for the three heavily affected central regions VI, VII and VIII (shaded in the map of Chile), according to the dummy EQ^2010^ (see Methods for a detailed description of this variable).

In this paper, we report evidence on a case where harsher environmental conditions have led to more social cohesion, as measured by a variety of indicators. Our study exploits regional and temporal variation in exposure to earthquakes across 15 regions and 346 *comunas* (the smallest administrative subdivision) in Chile. The regions and *comunas* we are comparing have very similar historical, cultural and traditional roots, share the same formal institutions and, unlike e.g. in [20], there are no pre-existing conflicts like organized political violence. They are, however, very differentially affected by earthquakes (Fig 1). Across several different indicators we find that social cohesion is higher in more affected regions. Our findings contribute to the literature on the impact of natural disasters more broadly and more specifically on social capital and social cohesion (the related literature is discussed in the last section).

## Earthquakes in Chile

Chile is a long narrow country extending approximately 4300 km from north to south. It is bordered by the South Pacific Ocean to the west and by the Andes Mountains to the east. The country lies on the boundary between the Nazca plate and the South American continent, one of the most seismically active areas in the World [21].

**Measuring Earthquake Impact.** The severity of an earthquake is traditionally measured by either its magnitude or its intensity. Magnitudes and intensities measure different characteristics of an earthquake. Earthquake magnitude quantifies the maximum energy released at the quake epicenter. Data provided by the National Seismological Center in Chile and the U.S. Geological Survey mostly report two magnitude scales: surface-wave magnitude (*Ms*, hereafter) and moment magnitude (*Mw*). We focus on these two throughout this study and report one or both, depending on their availability. Local magnitude, also known as Richter scale, is another measure of earthquake magnitude. All magnitude measures should yield approximately the same value for any given earthquake [22], but *Mw* tends to be higher than *Ms* for Chilean earthquakes (see below).

The problem of magnitude scales is that they do not necessarily reflect the impact of an earthquake on the surface nor how far-reaching a quake is. Earthquake intensity, by contrast, measures the impact of an earthquake on earth’s surface, on humans, and their structures. Intensities thus allow us to quantify the effect on an event in areas located far from the epicenter and the effect of the same event can differ across regions. The most commonly used intensity measure is the Modified Mercalli scale, which we also employ.

In what follows, we follow the notation in the literature on earthquakes. Magnitudes are written in Arabic letters, while intensities are denoted by Roman numerals. For example, an earthquake of magnitude 7.5 according to *Ms* at the epicenter is denoted by *Ms*7.5 and all earthquakes of magnitude 7 or higher as *Ms*7.0+. The notation is analogous for *Mw*. In case of the Modified Mercalli scale, we write, say, intensity VI in the Mercalli scale throughout.

Our analysis focuses on major earthquakes which we define to be of either *Ms*/*M*_*w*_ ≥ 7.0 or of intensity higher than or equal to VII in the Modified Mercalli Scale. Earthquakes with *Ms*8.5+ are referred to as *megaearthquakes*, but not treated differently in the analysis. A threshold of *Ms*7.0 is used because the National Seismological Center provides a series of historic important and/or destructive earthquakes of magnitude 7.0 or greater. Additionally, magnitudes higher than or equal to 7.0 are typically associated with intensities VII or greater. An intensity greater or equal to VII entails damages that go from “Damage negligible in buildings of good design and slight to moderate in well built ordinary structures” to “Damage Total” [22]. For the particular *Ms*7.0+ threshold, earthquakes are considered severe enough to entail important human and economic losses over large areas. Combining magnitudes and intensities to evaluate the impact of an earthquake is crucial because even areas at large distance from the epicenter can be severely affected. For example, even though the epicenter of the 2010 Maule megaearthquake was located 335 km south of Santiago de Chile, its aftershocks covered an area that extends from the coast to the trench for a length of over 600 km. As a result, Regions VIII and IX located to the south of Maule as well as regions VI and XIII (the Metropolitan Region) located to the north of the epicenter in Maule suffered intensities higher than or equal to VII in the Modified Mercalli Scale.

**History of Earthquakes in Chile.** Over the past six decades, several *Ms*7.0+ earthquakes have been registered in the area (Fig 1), including two *Ms*8.5+ megaearthquakes. The South Central Chilean subduction zone produced both megaearthquakes, the 1960 Valdivia earthquake and the recent 2010 Maule earthquake [21]. In May 1960, Valdivia, the capital of Region XIV located about 850 km south of Santiago de Chile, suffered the largest earthquake ever recorded worldwide since the beginning of instrumental seismology with a magnitude of *Ms*8.5 (*Mw*9.5) [23]. This megaearthquake caused important human and economic losses with approximately 660 fatalities, 717 missing persons, and ∼ 58000 houses entirely destroyed. Such a massive quake also caused important modifications to the coastal relief. Rock falls and landslides were observed in the Andes mountains, creating an artificial lake on the Río San Pedro, the outlet of Lake Rinihue [24].

In between the two megaearthquakes in 1960 and 2010, Chile has experienced several other important earth tremors. We briefly describe the most relevant in terms of impact. In March 1985, a *Ms*7.8 (*Mw*8.0) earthquake and its associated tsunami struck the south coast of Valparaíso (Region V). The epicenter was located about 125 km west of Santiago de Chile but the aftershock zone extended about 200 km in length north-south and was at least 100 km wide from east to west along the dip of the subducted Nazca plate [25]. This earthquake caused 176 deaths and about 2500 injured. Ten years later, in July of 1995, a *Ms*7.3 (*Mw*8.0) earthquake shook Antofagasta in the North of Chile (Region II), approximately 1400 km north of Santiago de Chile. In 2005, a *Ms*7.8 earthquake struck Tarapacá (Region I). The epicenter was located 49 km north of Pica and about 1,800 km north of Santiago de Chile. In November 2007, the northern region of Antofagasta suffered another earthquake of *Ms*7.5 (*Mw*7.7), the epicenter being located 43 km west of the *comuna* of María Elena about 1500 km north of Santiago de Chile. The 2007 shock caused two deaths, 4451 homes were damaged and 3012 destroyed.

In February 2010, Chile experienced its second megaearthquake in the last six decades, the 2010 Maule earthquake, which plays a crucial role in our *comuna*-level analysis. It represents the sixth largest earthquake ever recorded worldwide, with a magnitude of *Mw*8.8 [21]. This event originated 335 km south of Santiago de Chile, affected a zone that extends at least 450 km along the Chilean cost, and its aftershocks covered an area that extends from the coast to the trench for a length of over 600 km [26]. This illustrates that the impact of this event goes well beyond the epicenter. The 2010 Maule earthquake triggered a tsunami, which struck the coast nearest the epicenter within minutes after the massive quake. The earthquake and the tsunami killed at least 521 people, and damaged or destroyed ∼370000 homes, 3049 schools and 73 hospitals. The estimated economic losses were about 30 billion U.S. dollars, corresponding to 17% of Chilean GDP (see [27]).

As we can observe in Fig 1, Chile presents substantial cross-regional and temporal variation in the seismic activity. The geographical distribution of these earthquakes stretches almost across the entire country. We particularly exploit the 2010 *Mw*8.8 Maule earthquake in our statistical analysis, as this more recent event overlaps with available data on social cohesion. Fig 1 also shows the geographical distribution of the earthquake impact at the *comuna* level for three specific regions that were heavily affected by the 2010 Maule event (Regions VI, VII and VIII, shaded in the map of Chile).

## Methods

Our aim is to assess whether and how adverse environmental conditions affect indicators of social cohesion. Since earthquakes are exogenous to social cohesion and since their occurrence and strength are precisely recorded, they are a good measure of adverse environmental conditions. Our analysis is performed from two geographical perspectives, at the regional level and at the *comuna* level. Chile is divided in 15 regions. These regions are further divided into provinces, and the provinces into a total of 346 *comunas*. The reason to analyze both regions and *comunas* is the following. More information on social cohesion is available at the regional level. However, there are only 15 regions in Chile. *Comunas*, by contrast, provide more cross-sectional units but fewer variables are available at the *comuna* level.

Using data from the Chilean National Seismological Center and the US Geological Survey we construct several measures of exposure to earthquakes for both regions and *comunas*. At the regional level we consider two different measures. We construct a dummy variable ($EQ_{j}^{t}$) that identifies regions affected by a major earthquake at least once in the last three years, during the period 2003-2012. Region *j* is treated as affected ($EQ_{j}^{t}=1$) if within the last three years as measured from *t*, i.e. in years *t*, *t* − 1 or *t* − 2, (*i*) the epicenter of a *Ms*7.0+ earthquake was located there or (*ii*) the earthquake was felt in region *j* with an intensity equal to or higher than VII in the Modified Mercalli Scale.

The particular threshold of three years was selected because it entails a time span that matches the availability of our measures of social cohesion. There also exists a technical reason. If we select a threshold much larger than three years (e.g., ≥ 10), $EQ_{j}^{t}$ does not vary across time and its influence on the indicator of social cohesion is subsumed into the region fixed effects.

We also analyze a recency measure ($DISTEQ_{j}^{t}$) that measures when region *j* was last affected by a major earthquake. For regions unaffected by any major earthquake in the last 30 years we set this variable equal to 30. Compared to shorter-term measures, both $EQ_{j}^{t}$ and $DISTEQ_{j}^{t}$ are particularly relevant to understand more medium- or long-term effects that adversarial events may have on communities whose social norms and rules evolve under these conditions.

Since social cohesion data at the *comuna* level do not reach as far back in time, we focus on one single variable of exposure at the *comuna* level. $EQ_{c}^{2010}$ indicates whether *comuna* *c* was affected by the 2010 8.8-magnitude Maule earthquake or not. A *comuna* *c* is treated as affected if it satisfies at least one of the following three conditions: (*i*) it is identified by the seismological service as a comuna hit by the 2010 Maule earthquake (i.e., *comuna* *c* experienced an intensity greater than VI in the Mercalli scale); (*ii*) it suffered at least one fatal victim; (*iii*) it asked for economic aid. The variable $EQ_{c}^{2010}$ hence measures more short-term effects on social cohesion.

There is no consensus on how to measure social cohesion [28]. The OECD [29] categorizes social cohesion into five classes: (*i*) Life satisfaction, (*ii*) Trust, (*iii*) Social behavior, (*iv*) Suicide, and (*v*) Voting. We perform our analysis using eight indicators of social cohesion, one for each class (*i*), (*ii*), (*iv*) and (*v*), and four different indicators of social or anti-social behavior (all variables are summarized in Table 1; a more detailed description of all the indicators as well as their sources are provided in S1 Appendix). Our first indicator is Life Satisfaction (Life Sat), which measures the extent of agreement to the question “Are you satisfied with your life?” The second indicator is Trust, which measures the extent of agreement with the statement “Generally speaking most people can be trusted”. Both Life Sat and Trust are taken from the *Latinobarómetro*, a survey conducted every year in 18 different Latin American countries that gathers information on individuals’ attitudes and beliefs (S1 Appendix). The two variables measure the fraction of surveyed people in each area who report being satisfied or very satisfied with their life and those who claim to trust others, respectively. We have four indicators for social or anti-social behavior (category (*iii*)). The first one is charitable giving (Charity), which is viewed as part of social behavior [29] and defined as the average amount of voluntary donations per person to a yearly fund-raising event broadcasted on television, named *Teletón*, that aims to raise funds to help children with disabilities. Our second indicator of social behavior is volunteering (Volunteering). This variable measures the percentage of people in an area who self-report having done any voluntary work in the Chilean household survey *CASEN*. The third indicator is criminality (Crime) which we expect to be inversely related to social behavior. This index encompasses crimes both reported to the police by the citizens and discovered by any police officer per each 100000 inhabitants. The index considers various forms of crimes, such as aggravated assault, murder, rape, robbery, burglary, motor vehicle theft, etc. Our fourth indicator is corruption (Corruption), also considered inversely related to social behavior [29]. To construct this variable we make use of a national survey that asks citizens whether they or any member of their family have been solicited for bribes by some public officer. The index shows the percentage of households that were solicited for bribes in a particular year. For category (*iv*) we use an index of suicides (Suicides) that measures the number of suicides per 100000 inhabitants according to national statistics. Finally, electoral participation (Voting) is our eighth indicator, which measures the percentage of people who showed up at the polls.

**Table 1:** Variables. Definitions, means, standard deviations and numbers of observations for key variables. See S1 Appendix for further details regarding these variables).

		Mean	SD	N	Years	Reg/Com
	**Earthquake exposure**					
$EQ_{j}^{t}$	= 1 if affected by a major eq. in last 3y	0.175	0.382	120	05-12	Region
$EQ_{j}^{2010}$	= 1 if affected by the 2010 eq.	0.217	0.412	960	09-11	*Comuna*
$DISTEQ_{j}^{t}$	years (2 digits) to last major eq.	20.86	13.13	120	05-12	Region
POST	= 1 if *y* > 2010	0.333	0.472	960	09-11	*Comuna*
	**Social Cohesion**					
Life Sat	% people very satisfied or satisfied with life	0.689	0.205	367	08-11	*Comuna*
Trust	% people expressing high level of trust	0.162	0.209	367	08-11	*Comuna*
Charity	donation to Teleton (CLP per capita)	700.98	241.10	75	07-12*	Region
Volunteering	% population engaged in volunteering	0.362	0.047	30	09,11	Region
Volunteering	% population engaged in volunteering	0.387	0.096	640	09,11	*Comuna*
Voting	% participation in local elections	0.381	0.064	30	08,12	Region
Crime	crimes per 100,000 inhabitants	3175.86	599.76	105	05-11	Region
Crime	crimes per 100,000 inhabitants	2770.84	1687.54	960	09-11	*Comuna*
Corruption	% solicited for bribes by public servants	0.006	0.010	114**	05-12	Region
Suicides	suicides per 100,000 inhabitants	12.76	3.05	105	05-12	Region
Suicides	suicides per 100,000 inhabitants	14.80	12.03	960	09-11	*Comuna*

eq.= earthquake, y = year, Reg = region, Com= *comuna*, CLP = Chilean peso,

*Not available for 2009 due to presidential elections.

**Chile was reorganized from 13 to 15 regions in 2007, leading to a loss of 6 observations.

The units and time spans of all these variables are summarized in Table 1. For expositional purposes, we informally classify these eight variables into positive (Charity, Volunteering, Voting, Life Sat, Trust) and negative (Crime, Corruption, Suicides) according to their relation with social cohesion. For instance, more volunteering is associated with more cohesive societies while higher crime is considered a sign of lower social cohesion. Finally, we would like to mention that due to the lack of available data only Volunteering, Crime, and Suicides are available at both regional and *comuna* level. Charity, Voting, and Corruption are not disaggregated at the level of *comunas*, whereas Life Sat and Trust can solely be constructed at the *comuna* level due to the sampling strategy of *Latinobarómetro*, which focuses intensively on specific regions.

Since regional differences in social cohesion could come about by a number of factors other than exposure to earthquakes, it is imperative to account for this possibility. To do so we use two different approaches: (i) Fixed Effects regressions which control for some key indicators (gender ratio, income, poverty, Gini coefficient, and net migration rate as well as year dummies) and (ii) Principal Component Analysis (reported in S1 Appendix) which controls for a larger number of regional characteristics. All variables are standardized to mean zero and standard deviation (SD) one in the regressions. Statistical approaches are described in more detail in Results. The complete information on the data is provided in S1 Appendix.

## Results

**Regions.** We start this section by discussing our regional level results. We estimate the following fixed-effects models:

$$\begin{array}{c} y_{jt}=\alpha_{j}+\beta {\text{Earthquake}}_{jt}+\gamma X_{jt}+\epsilon_{jt}, \end{array}$$ (1)

where *y*_*jt*_ is an indicator of social cohesion in region *j* at time *t*, Earthquake is our measure of earthquake exposure (either EQ or DISTEQ) and **X**_*jt*_ is a vector of controls. *α*_*j*_ is a region fixed effect, which can be arbitrarily correlated with the controls **X**_*jt*_. This term captures unobserved variables that do not vary over time, such as e.g. cultural or geographical factors. A key implication of this approach is that, since the unobserved variables do not change over time, any change in the outcome variable cannot be attributed to these fixed characteristics. The coefficient of interest is *β*, which shows how earthquake exposure affects the social cohesion indicator in question on average across affected regions. Coefficients *γ* are not reported explicitly in Table 2 but can be found in S1 Appendix.

**Table 2:** Regional analysis. Regional fixed effects regressions of the effect of earthquakes exposure on positive (columns (1-6)) and negative (columns (7-12)) indicators. Controls include lagged Gini coefficient, migration rate, (log of) income, poverty, gender ratio and year dummies and are explicitly reported in S1 Appendix. Standard errors clustered at the regional level. Significance level (***) 1%; (**) 5% and (*) 10%.

	Unit of observation: Chilean regions					
	Charity		Volunteering		Voting	
	(1)	(2)	(3)	(4)	(5)	(6)
**Variable**						
EQ	0.34**		0.154		0.419*	
	(0.132)		(0.243)		(0.232)	
DISTEQ		−0.128**		−0.599***		−0.185
		(0.058)		(0.172)		(0.226)
Observations	56	71	28	30	28	28
Regions	15	15	15	15	15	15
R-squared	0.481	0.479	0.912	0.700	0.934	0.837
Region Fixed Effects	YES	YES	YES	YES	YES	YES
	Unit of observation: Chilean regions					
	Crime		Corruption		Suicides	
	(7)	(8)	(9)	(10)	(11)	(12)
Variable						
EQ	−0.452**		−0.099		−0.258	
	(0.188)		(0.175)		(0.328)	
DISTEQ		0.146**		0.009		0.261***
		(0.063)		(0.072)		(0.084)
Observations	54	84	69	99	54	84
Regions	15	15	15	15	15	15
R-squared	0.543	0.658	0.139	0.091	0.165	0.157
Region Fixed Effects	YES	YES	YES	YES	YES	YES

Odd regressions in Table 2 show how regions exposed to at least one earthquake in the previous 3 years ($EQ_{j}^{t}=1$) differ from unaffected regions in terms of our six indicators of social cohesion. Being effected by an earthquake in the last three years is associated with higher giving to charity (column (1)). Giving increases by 34% of a SD more per capita. This amounts to ≈ 82 Chilean pesos (at 2007 real prices ≈ 0.2 US dollars) per each person living in the region. If, say, 10% of the population of a region participate in the charity event this number increases to 2 US dollars per person. In addition, people in affected regions are 42% (of a SD) more likely to vote (column (5)). The effect of exposure on Volunteering goes in the expected direction but is not statistically significant at the regional level.

Negative indicators, by contrast, decrease with exposure to earthquakes. Crime rates are 45% of a SD lower in affected compared to unaffected regions (column (7)). This amounts to approximately 271 crimes per 100000 inhabitants. Although the estimated coefficients for corruption and suicides show the expected direction, they are not statistically significant (columns (9) and (11)). Overall, the results suggest that adversarial conditions create social cohesion. People in affected regions display more positive and less negative indicators. As one way to check that our results are not caused by factors unrelated to earthquakes we perform the following Placebo test. We first randomly distribute the value of $EQ_{j}^{t}=1$ to different regions, maintaining the number of instances $EQ_{j}^{t}=1$ appears in the observed data, and reestimate our models to see whether a random distribution of earthquake exposure generates the same estimates as the observed exposure. We repeat this exercise 10000 times and record how often such a random distribution of exposure reproduces our results (see S1 Appendix for details). If the effect is driven by exposure to earthquakes as opposed to other more mechanical forces, we should obtain an average null effect under this *fake* earthquake exposure. Our test shows that the probability of obtaining the result in Table 2 by pure chance is ≈ 3.1%, suggesting that it is relatively unlikely that our results are accidental.

To understand better the nature of the above estimates we analyze, by means of our variable DISTEQ, whether social cohesion erodes if there is a prolonged period without earthquakes. The results, reported in even columns in Table 2, suggest that there is erosion of social cohesion after periods in which environmental conditions are less adverse. For instance, on average people give roughly 31 Chilean pesos less to Charity approx. 13 years after the last earthquake (≈1 SD of DISTEQ), whereas the corresponding reduction in Volunteering is $\left|\hat{\beta }\right|\times SD_{\text{Volunt}}\;=\;0.599\;\times \;0.047=2.8\%$. There are no statistically significant effects on voter turnout. Negative behavior, by contrast, is on the increase after periods of tranquility. An increase of 1 SD in DISTEQ (slightly more than 13 years) leads to an increase of 464 crimes per 100000 inhabitants on average and to an increase of three suicides per 100000 inhabitants. The effect on corruption is not statistically significant, but goes in the expected direction. All regional-level results are robust to performing the principle component analysis that allows for controlling for a larger set of regional characteristics (S1 Appendix).

**Comunas.** At the *comuna*-level we focus on the 2010 *Mw*8.8 Maule earthquake, one of the biggest earthquakes in Chilean history and the sixth largest earthquake ever recorded worldwide. We use the following difference-in-differences (diff-in-diff, hereafter) approach, in which we compare the affected and unaffected *comunas* before and after the 2010 Maule earthquake. The estimated model is

$$\begin{array}{c} y_{ct}=\alpha +\beta_{1}{\text{POST}}_{t}+\beta_{2}{\text{EQ}}_{c}^{2010}+\beta_{3}{\text{POST}}_{t}\times {\text{EQ}}_{c}^{2010}+\gamma X_{ct}+\epsilon_{ct}, \end{array}$$ (2)

where POST_*t*_ is a dummy variable that equals 1 if *t* ≥ 2011 (the year after the earthquake) and 0 for years before the earthquake, $EQ_{c}^{2010}$ is a dummy variable that equals 1 if *comuna* *c* was hit by the Maule earthquake in 2010, and $POST\times {EQ}_{c}^{2010}$ is the interaction between the two. Since Life Sat and Trust were collected six months after the February 2010 Maule earthquake, estimations with these indicators set POST_*t* = 1_ if *t* ≥ 2010 and 0 otherwise. For Volunteering, Crime and Suicides there are no available data for 26 comunas (out of 346) and we only have reliable data on Life Sat and Trust for 98 *comunas*.

Table 3 reports the results of the diff-in-diff regressions. The estimated *β*_1_’s reflect the average increase or decrease in the indicator between the period before and after the 2010 Maule event for the unaffected *comunas* and the coefficients *β*_2_ measure the difference between affected and unaffected *comunas* before the 2010 Maule earthquake. Our main interest lies in the parameter *β*_3_ that estimates the average difference in the evolution of the indicator between affected and unaffected *comunas* from before the event to post-earthquake.

**Table 3:** *Comuna* level difference in differences regressions on the effect of earthquakes exposure on life satisfaction, trust, volunteering, crime and suicides. Controls include lagged Gini coefficient, migration rate, (log of) income, poverty, gender ratio and year dummies and are explicitly reported in S1 Appendix. Standard errors are clustered at the province level (S1 Appendix). Significance level (***) 1%, (**) 5% and (*) 10%.

	Unit of observation: Chilean *comunas*				
	Life Sat.	Trust	Volunteering	Crime	Suicides
	(1)	(2)	(3)	(4)	(5)
Variable					
POST	−0.813***	−0.157	0.465***	0.186*	0.111
	(0.245)	(0.221)	(0.101)	(0.109)	(0.096)
$EQ_{c}^{2010}$	−0.333	−0.277	−0.404**	0.134	−0.146*
	(0.251)	(0.272)	(0.155)	(0.098)	(0.075)
$POST\times {EQ}_{c}^{2010}$	0.742***	0.110	0.265***	−0.162**	−0.048
	(0.268)	(0.183)	(0.128)	(0.062)	(0.111)
Observations	227	227	640	960	960
*Comunas*	98	98	320	320	320
R-squared	0.204	0.04	0.350	0.484	0.076

Prior to the 2010 Maule earthquake (POST = 0) affected communities display 40% of a SD lower rates of volunteering compared to unaffected *comunas*. This changes after the 2010 Maule earthquake, where affected *comunas* display 27% higher rates of volunteering compared to others. Life satisfaction rates are no different between affected and unaffected *comunas* pre-earthquake, but after the Maule earthquake affected *comunas* exhibit 74% higher rates of life satisfaction compared to unaffected *comunas*. Likewise, crime rates are no different before the earthquake, but 16% lower in affected *comunas* post-earthquake. The diff-in-diff analysis does not yield statistically significant results for Trust and Suicides. As for the former, even though this variable is standard across a number of household surveys worldwide, it has been criticized as a measure of trust. In particular, the authors in [30] note that it predicts trustworthiness much better than trust.

We performed several robustness tests for these findings (see S1 Appendix). First, a *comuna*-level principal component analysis yield qualitatively similar results. Second, a Placebo test similar to the one presented above reveals that the probability of obtaining our *comuna* results by chance is only ≈ 0.17%. Third, an alternative Placebo test with an artificial event year (2013 instead of 2010) shows null effects as well. Both tests suggest strongly that the 2010 Maule earthquake is the reason behind the detected differing evolution of social-cohesion indicators between the affected and unaffected *comunas*.

Finally, S1 Appendix contains individual-level regressions for Volunteering and two particular aspects of Crime. The estimates are consistent with our previous *comuna*-level results and demonstrate that they are robust to migratory movements, which suggests that our results are not driven by systematic movements of the population from and to (un)affected regions.

## Discussion

Increased exposure to earthquakes seems to be consistently associated with higher levels of positive and lower levels of negative social cohesion indicators both across 15 Chilean regions and 320 Chilean *comunas*. People in more affected regions give more to charity, are more likely to engage in volunteering and vote, are less likely to engage in crime, and seem to be more satisfied with their lives. Since these differences erode over time, it further points to an interaction between earthquake exposure and social cohesion.

This shows that, apart from the significant and well studied economic consequences of natural disasters [31–35], there is also a substantial impact on social cohesion, which may go well beyond short-run responses [36, 37]. People seem to compensate for worse environmental conditions by being more cooperative. Combined with the right technology and institutions, social cohesion can lead to efficiency gains in production that could be part of the explanation why natural disasters sometimes lead to improved economic outcomes [35]. To the extent that direct economic effects operate more in the short run while improved social cohesion tends to last longer and possibly creates positive indirect economic effects, disasters may cause negative economic effects in the short run but positive effects in the long run. Such trade-offs may, partially explain why such mixed results have been found in terms of economic effects of natural disasters [35, 38].

Our results complement the emerging literature documenting positive effects of natural disasters. Toya and Skidmore [39] provide a cross-country analysis of trust and report positive association between trust and disaster occurrence, including earthquakes. Few studies use experimental methods and document short-term positive impacts of these events in a particular area on trust [40, 41] or cooperative behavior [36, 37], but Fleming et al. [42] observe no effects of the 2010 Maule earthquakes on trust and negative effects on reciprocity in the immediate aftermath of the event. Dussaillant and Guzmán [43] report a positive impact of the 2010 Maule earthquake on trust in the medium run. The idea that adverse events may build social cohesion is also mentioned in the World Happiness Report 2015 [44]. Our contribution to this literature is twofold. First, we provide a more systematic investigation of the effects in that we cover a larger variety of indeces of social cohesion. Moreover, we focus on one country with relatively homogenous culture and formal institutions and, unlike the experimental studies, our approach allows to explore the effects at the level of the whole country and at different geographic units.

Furthermore, our findings add to the resilience research that has explored how communities develop strategies to deal with (frequent) natural disasters. The need for a better understanding of the linkages between ecosystems and human societies has been pointed out by [45]; see [46] for a thorough discussion of these issues. In the context of earthquakes, resilience has been categorized into four groups: technical, organizational, social, and economic [47]. Our research provides evidence on increased social resilience. Unlike the resilience achieved through government programmes, such as e.g. those included in the 2000 Disaster Mitigation Act in the United States [47], the increased social cohesion we identify in this paper seems to emerge endogenously without outside interventions in the affected communities.

Our results support the “environmentalist hypothesis” that environmental conditions shape social organization [8, 9, 11, 12]. Previous research has identified a positive relation between adversarial environmental conditions and within-group conflicts (e.g. [17, 20]). It is not clear how social cohesion and conflict are related. Not all forms of conflict are indicative of low social cohesion as pointed out by Bowles [48], conflict could simply respond differently to adverse environmental conditions than the dimensions of social cohesion addressed here, and not all studies on conflict and adverse conditions find a positive relation [32, 49].

Even though our data have unusually rich variation in earthquake exposure, our study still presents several limitations. The data on social cohesion indicators are still limited at smaller geographical scales. Understanding the mechanism underlying the interaction between environmental conditions and social cohesion would greatly benefit from complementing our analysis with extensive experimental analysis of social cohesion indicators. Future research is needed to uncover the precise social mechanisms behind our findings. In this direction, one recent study has shown that, after being exposed to a hurricane, people seem to create networks associated with higher social cohesion [50]. One could also ask whether our findings would be replicated in other countries. One difficulty in doing so is that few countries in the world exhibit the type of spatiotemporal variation in exposure to natural disasters Chile does.

## Supporting information

S1 Appendix Online supplementary material. Online Supplementary Material contains additional information concerning our data and analysis.
(PDF)
