# Supplementary material for: Natural disasters and indicators of social cohesion
Source: PLoS One. 2017 Jun 7;12(6):e0176885. doi: 10.1371/journal.pone.0176885 (PMC5462365; doi:10.1371/journal.pone.0176885)
Supplement: S1 Appendix — Online Supplementary Material contains additional information concerning our data and analysis. (PDF) [file pone.0176885.s001.pdf]

Natural Disasters and Indicators of Social Cohesion Online Supplementary Material

Aitor Calo-Blanco*
University of A Coruña

Jaromír Kovářík†,‡
University of the Basque Country and CERGE-EI

Friederike Mengel§
University of Essex

José Gabriel Romero¶
University of Santiago de Chile

March 31, 2017

Additional Information on Data and Variables

A.1 Regional Level Seismic Data. We use seismic data provided by the National Seismological Center (*www.sismologia.cl*) and the US Geological Survey (*http : //earthquake.usgs.gov*). At the regional level we then construct two different measures of the exposure to earthquakes:

1. $\mathtt{EQ}_j^t$ is a dummy that takes the value 1 if region j has been affected by at least one earthquake within the last 3 years as measured from t, i.e. in years $t, t-1$ and $t-2$. A region is treated as affected ($\mathtt{EQ}_j^t$=1) if the epicenter of an earthquake of magnitude Ms higher than 7 was located there and/or the intensity (measured by the Modified Mercalli Scale) the region experienced was equal to or higher than VII. We use earthquake data from 2003-2012 to correspond with the availability of our measures of social cohesion. In addition, for higher threshold values EQ_j^t does not vary across time and the effect of earthquake exposure is subsumed into the fixed effects.

2. $\mathtt{DISTEQ}_j^t$ denotes the distance in years between period t and the last year that region j was affected by an earthquake. For regions that have not suffered any earthquake in the last 30 years, we set this variable to 30.

Figure 1 in the main text illustrates the regional variation in earthquake exposure and visualizes the temporal variation in earthquakes, generating variation in the measure $\mathtt{DISTEQ}_j^t$.

*Dpto. de Análisis Económico y Admon. de Empresas, Facultad de Economía y Empresa, Campus de Elviña s/n, 15071 A Coruña, Spain (aitorcalo@gmail.com).

†Dpto Fundamentos Análisis Económico I & Bridge, Universidad del País Vasco/Euskal Herriko Unibersitatea, Av. Lehendakari Aguirre 83, 48015 Bilbao, Spain (jaromir.kovarik@ehu.es).

‡CERGE-EI, a joint workplace of Charles University Prague and the Economics Institute of the Czech Academy of Sciences, Politických vězňů 7, 111 21 Prague, Czech Republic.

§Department of Economics, University of Essex, Wivenhoe Park, Colchester CO4 3SQ, United Kingdom (fr.mengel@gmail.com).

¶Departamento de Economia, USACH, Alameda 3363, Santiago, Chile (gabriel.romero@usach.cl).

It should be pointed out that if we lower the threshold to $Ms/Mw6.0+$ only one previously unaffected region becomes affected (using the $\texttt{EQ}_j^t$ dummy); if we increase it to $Ms/Mw8.0+$ we exclude instances of earthquakes with important human and economic losses and substantially reduce variation in our earthquake indicators.

A.2 *Comuna* level seismic data. Some of our variables that capture social cohesion are expressed at the level of Chilean *comunas*. To identify the affected *comunas*, we complement the information of the National Seismological Center with that of the Legal Medical Service (LMS) of the Ministry of Justice (*http : //www.sml.cl/sml/*) and the Chilean Association of Municipalities (*http : //www.munitel.cl/*). The reason is that the seismological service does not always provide information of the affected areas at the *comuna*-level. We define two *comuna*-level measures of earthquake exposure:

1. $\texttt{EQ}_j^{2010}$ is the dummy variable that equals 1 if *comuna* j: (i) is identified by the seismological service as a *comuna* hit by the 2010 Maule earthquake (i.e., a *comuna* that suffered an intensity greater than or equal to VII in the Mercalli scale), and/or (ii) had at least one fatal victim, and/or (iii) asked for economic aid.

2. $\texttt{DISTEQ}_j^t$ measures the distance in years between period t and the last year that *comuna* c was hit by an earthquake.

A.3 Social Cohesion. Defining and measuring social cohesion is difficult. As noted by [**S1**], there is a "proliferation of definitions of social cohesion that have proved difficult to combine or reconcile" (p. 409). We focus on measures of positive and negative behavior proposed by the OECD [**S3**].

Positive Behavior. Our variables `Life Satisfaction` and `Trust` are obtained from the 2008, 2009, 2010, 2011 and 2013 waves of the *Latinobarómetro*, an annual survey that gathers information on attitudes and beliefs of individuals from 18 Latin American countries (more information and the data are available at *http://www.latinobarometro.org/*). Since we are interested in studying the effects of earthquake exposure on indicators of social cohesion in Chile, in case of these two variables we select Chile and eliminate all non-Chilean citizens. This reduces our sample to 1159 individuals in 2008, 1183 in 2009, 1173 individuals in 2010, 1185 individuals in 2011 and 1177 individuals in 2013. We group individuals according to their *comuna* of residence. We have observation for only 98 Chilean *comunas*. Since we have too few observations for several regions, reliable data are available for much less than 15 regions and `Life Sat` and `Trust` are therefore only used at the *comuna* level. Using less than 15 cross-sectional units is too few to make any meaningful analysis at the level of regions.

To construct the variable `Life Sat` we use the following question (Q27ST in 2008, Q1ST in 2009-2013): *"In general, would you say you are satisfied with your life? Would you say you are ...?"*. The possible answers are: (1) Very satisfied, (2) Fairly satisfied, (3) Not very satisfied and (4) Not satisfied at all. $\texttt{LifeSat}_{j,t}$ measures the percentage of people living in *comuna* j, in period t, that choose options (1) or (2). For variable `Trust` we use the following question (Q21WVSST in 2008, Q58ST in 2009, Q55ST in 2010, Q25ST in 2011 and Q29STGBS in 2013): *"Generally speaking, would you say that you can trust most people, or that you can never be too careful when dealing with others?"*. The possible answers are: (1) One can trust most people and (2) One can never be too careful when dealing with others. $\texttt{Trust}_{j,t}$ measures the percentage of people living in *comuna* j, in period t, that choose (1).

Our variable `Charity` is obtained from the *Teletón*, a yearly fund-raising event broadcasted on television in Chile since 1978 (*www.teleton.cl*). This charity event collects voluntary donations across the whole country in order to raise funds to help children with disabilities who are treated at health-related organizations of the *Fundación Teletón*. We use regional data on contributions to the *Teletón* between 2007 and 2012. It is important to note three features of this charity event. First, data corresponding to previous editions are not available at a regional level. Second, for this dimension of social cohesion, information is only available at the level of regions. Third, in 2009 and 2013 the event did not take place because the presidential elections were held. Additionally, we would like to stress that natural disaster relief was never a charity aim of the selected sample. There was a special *Teletón* event for the victims of the 2010 Maule earthquake, different from the standard 2010 edition, that we excluded from our sample.

For volunteering we use data from the 2009, 2011 and 2013 waves of the CASEN [*Encuesta de Características Socioeconómicas nacionales* (Survey of national socioeconomic characteristics) available at *www.ministeriodesarrollosocial.gob.cl/casen*]. Since 2009 CASEN contains a question about volunteering. We use the following question (*t18a* in 2009 and *r9* in 2011 and 2013): *"Are you doing any voluntary work?"*. The possible answers are: (1) Neighborhood organizations; (2) Sport club; (3) Religious organizations (this answer explicitly excludes activities such as prayer-activity, going to mass and the like); (4) Art groups; (5) Cultural groups; (6) Student/youth centres; (7) Women associations; (8) Associations for elderly people; (9) Volunteer groups; (10) Self-health groups; (11) Political party; (12) None. Volunteering does *not* include relief efforts directly related to the consequences of earthquakes. The only category that might subsume such effects is category (9) and the percentage of people that tick the corresponding box is 0.42%, 0.45% and 0.39%, respectively, in the 2009, 2011 and 2013 CASEN waves. Our variable $\texttt{Volunteering}_{j,t}$ measures the percentage of people living in region (*comuna*) j, in period t, that choose any option from (1) to (10). We have observation for 15 Chilean regions and for 320 out of 346 *comunas*. In Table 11 below we also exploit individual-level variation in volunteering.

To measure electoral participation we use the number of persons who showed up at polls in the 2008 and the 2012 elections of mayors and council members. These data come from the Chilean Electoral Service (*Servicio Nacional Electoral*, available at *www.servel.cl*). The resulting variable $\texttt{Voting}_{j,t}$ measures the percentage of people who showed up at the polls in region j at period t.

Negative behavior. To construct the variable `Crime` we use official data on criminal activity provided by the Chilean government. Since 2005 the Ministry of the Interior (*Ministerio del Interior y Seguridad Pública*) prepares and publishes crime rates classified by crime types according to their social impact (*"Tasa de casos policiales por delitos de mayor connotación social"* in Spanish; see *http : //www.seguridadpublica.gov.cl*). This index encompasses crimes both reported to the police by the citizens and discovered by any police officer per each 100000 inhabitants. The episodes the index includes vary from violent crimes, such as like aggravated assault, murder, rape, robbery, to property crimes such as burglary, motor vehicle theft etc. These data are available at the level of both regions and *comunas* for the period 2005-2011.

We study two other measures of negative behavior: suicides and corruption. The data on suicides come from the Department of Statistics of the Ministry of Health (*www.deis.cl*) for 2005-2011. The variable $\texttt{Suicides}_{j,t}$ measures the rate of suicides per 100000 inhabitants in region/*comuna* j in period t. The data on corruption are from the Citizen Safety Survey (*Encuesta Nacional Urbana de Seguridad Ciudadana* from the Ministry of the Interior). Individuals are asked whether they, or any member of their family, were solicited for bribes by some public office. We have observations for 2005-2012 at the regional level. $\texttt{Corruption}_{j,t}$ measure the percentage of households solicited for bribes in region j at year t. This variable exhibits very little variation though. This may explain

why we detect no association between Corruption and Earthquake in our regressions.

A.4 Controls. We control for a large number of socio-economic characteristics from the 2006, 2009, 2011 and 2013 waves of the National Socioeconomic Survey (CASEN), the 2005-2009 waves of the Supplementary Survey of Income (ESI) and the 2010-2012 New Supplementary Survey of Income (NESI), available at *www.ine.cl*.

From CASEN at the level of both regions and *comunas* we use average years of schooling (Schooling), the percentage of poor people, Poverty, the percentage of females (Women) and migration between regions (Net Migration Rate). This variable measures migration flows between Chilean regions/*comunas*. CASEN asked individuals in which *comuna* their mother lived when they were born. With this information we compute "domestic immigration" and "domestic emigration." By the former we refer to the percentage of Chilean people who live in a different region from the one they were born. Domestic emigration measures the percentage of Chilean people who left their region of birth. $\texttt{Net Migration Rate}_{j,t}$ is the difference between domestic immigration and domestic emigration at year t in region/*comuna* j. It aims to control for possible biases due to the endogenous composition of Chilean regions.

We stress that the years of the CASEN survey do not perfectly match the years of the dependent variables Charity, Crime, Suicides and Corruption. To solve this discrepancy we compute the missing values using the annual rate of increase between periods 2006-2009, 2009-2011 and 2011-2013.

The variable Income is from the ESI and NESI. Because the ESI data is hard to compare with the NESI series in terms of non-labor income [**S2**], our definition of income includes labor income (i.e., salaries and wages, monetary or in kind royalties, commissions and income of professionals and self-employed) and pensions and widow's pensions. In particular, we compute per capita household income (income, hereafter) and the corresponding Gini coefficient. All monetary variables are expressed in Chilean Pesos (CLP) at 2007 real prices. Finally, data on population size are obtained from the National Institute of Statistics (INE).

Since earthquakes do affect economic variables such as income, poverty or migration, the variables $\texttt{EQ}^t$ and $\texttt{DISTEQ}^t$ could explain some of these variables at time t. To mitigate such effects we control for lagged variables. More precisely, since an earthquake at time t, $t-1$, or $t-2$ cannot affect income, poverty, Gini and migration in $t-3$, the controls are lagged three periods whenever $\texttt{EQ}^t$ is applied. For our recency measure $\texttt{DISTEQ}^t$ the controls come from $t-1$.

For the variables *Life Satisfaction* and *Trust* we use additional controls from the *Latinobarómetro* data base. Since in the *Latinobarómetro* individuals are asked about their ideological position, we control for this observable characteristic by using $\texttt{Left}_{j,t}$, $\texttt{Right}_{j,t}$ and $\texttt{None}_{j,t}$. $\texttt{Left}_{j,t}$ measures the percentage of people living in *comuna* j, in period t, that place themselves on the left in the left-right axis, $\texttt{Right}_{j,t}$ is the percentage of people in *comuna* j, at t, who place themselves on the right and $\texttt{None}_{j,t}$ measures the percentage of people who do not place themselves ideologically on the left or on the right. We also use the average age of individuals, the percentage of people with Low, Medium and High education level. Finally, we use the variable $\texttt{High} - \texttt{Income}_{j,t}$ that measures the percentage of people who cover their needs in a satisfactory manner with their total income family.

Table 1 in the main text summarizes the earthquake-related variables and the indicators of social cohesion. Tables 1 and 2 provides additional information regarding these variables as well as the descriptive statistics of the control variables.

Additional Tables and Results

	All Regions	`EQ`= 1	`EQ`= 0	Δ_{1-0}	ranksum test
Mean income (CLP)	155741	150632	156894	-6262	$p = 0.921$
Median income (CLP)	96315	98873	96867	2006	$p = 0.883$
Gini Index	0.533	0.541	0.531	0.01	$p = 0.223$
Poverty	0.143	0.145	0.143	0.002	$p = 0.969$
Schooling (years)	10.00	10.04	9.995	0.05	$p = 0.796$
Women	0.52	0.521	0.513	0.008***	$p = 0.008$
Net Migration Rate	-0.025	-0.036	-0.023	-0.013	$p = 0.514$
Population (mill)	1.08	1.92	0.91	1.004***	$p = 0.001$
`Charity`	701	764	659	105	$p = 0.467$
`Volunteering`	0.362	0.377	0.356	0.021	$p = 0.876$
`Voting`	0.381	0.374	0.383	-0.009	$p = 0.641$
`Crime`	3175	3388	3133	255**	$p = 0.034$
`Suicides`	12.76	11.78	12.93	−1.15	$p = 0.145$
`Corruption`	0.006	0.005	0.006	0.001	$p = 0.686$
Earthquake Magnitude	-	8.03	-	-	
`DISTEQ`	-	1.71	-	-	
`EQ`	0.216	-	-	-	
Earthquake frequency	-	2.08	-	-	

Table 1: Descriptive statistics: units of observation Chilean regions. Δ_{1-0} denotes the difference in means between affected and unaffected regions. The last column test for statistical significance using two-sided ranksum tests.

Table 1 summarizes some descriptive statistics at the regional level, separately for affected (`EQ`= 1) and unaffected (`EQ`= 0) regions. Apart from the differences in our variables of interest, affected regions tend to be more populated and to have relatively more women.

Table 2 shows descriptive statistics at the *comuna* level , separately for affected ($\texttt{EQ}^{2010} = 1$) and unaffected ($\texttt{EQ}^{2010} = 0$) *comunas.* All numbers are averages across the years 2009 (`POST`=0) and 2011 (`POST`=1).

	All	$\texttt{EQ}^{2010} = 1$	$\texttt{EQ}^{2010} = 0$	Δ_{1-0}	ranksum test
Mean Income	158830	132914	165954	-33040***	$p = 0.00$
Gini Index	0.44	0.43	0.44	-0.01***	$p = 0.02$
Poverty	0.164	0.185	0.158	0.027***	$p = 0.00$
Schooling	9.5	9.39	9.55	-0.16	$p = 0.27$
Net migration rate	-0.06	-0.12	-0.04	-0.08***	$p = 0.01$
Women	0.51	0.51	0.51	0.00	$p = 0.57$
Low-Education	0.244	0.228	0.250	-0.022	$p = 0.299$
Medium-Education	0.538	0.563	0.529	0.034	$p = 0.12$
High-Education	0.218	0.209	0.221	-0.012	$p = 0.832$
Left	0.534	0.595	0.513	0.082***	$p = 0.004$
Right	0.212	0.223	0.207	0.016	$p = 0.282$
None	0.206	0.139	0.23	-0.091***	$p = 0.001$
High-Income	0.516	0.458	0.537	-0.079***	$p = 0.001$
Age	43.49	43.53	43.47	0.06	$p = 0.676$
$\texttt{EQ}^{2010}$	0.22				
`DISTEQ`	30.39				
`Life Sat`	0.688	0.681	0.691	-0.01	$p = 0.72$
`Trust`	0.162	0.134	0.172	-0.038***	$p = 0.08$
`Volunteering`	0.38	0.39	0.38	0.01	$p = 0.32$
`Crime`	2621.87	2518.62	2650.25	-131.63	$p = 0.56$
`Suicides`	14.94	14.56	15.04	-0.48	$p = 0.84$

Table 2: Descriptive statistics collapsed at level of Chilean *comunas.*

Regional Regressions Tables 3 and 4 report the estimates of the Fixed Effect model (1) (see Results in the main text). Remember that some controls are lagged three periods whenever $\texttt{EQ}_j^t$ is

applied, and they are lagged one period for our recency measure $\texttt{DISTEQ}_j^t$. Tables 3 and 4 correspond to the estimations reported in the main text but coefficients of controls are included.

Many controls in the vector $\mathbf{X}_{it}$ will tend to be correlated (such as e.g. income and poverty). As an additional robustness check, and to control for multi-collinearity problems, we also performed a principal components analysis. We apply parallel analysis to filter out the most important part of the variance from *all* the observed measures and to determine the number of components. There is a total of nine components initially (the variables from Table 1 as well as the variable `year`). The analysis suggests that three components should be retained as the eigenvalues of the first three components are higher than one. In total, these three components account for 76% of the variance of the eight included variables. These three components are used in the principal component estimations. The results of these estimations are reported in Tables 5 and 6.

***Comuna*-level regressions** At the *comuna* level we use the difference in differences estimator in expression (2) in Results, where we compare affected and unaffected *comunas* before and after the 2010 Maule earthquake. Some control variables are again lagged. Table 7 reports the estimates. As with the regional level regressions we also conduct a principal component analysis for the *comuna*-level data. The results are reported in Table 8 and show qualitatively similar results as the regressions reported in Table 7.

Placebo Test I We conduct two types of placebo tests. We start by using a "fake" treatment group, where we assign $EQ = 1$ randomly to regions/*comunas*. We perform this experiment 10,000 times. If the effect is driven by exposure to earthquakes as opposed to other more mechanical forces we should see a null effect under this specification.

Regions. For the regional level regressions we assign each region a random number n_r^i, with $i = \{1, 2, 3\}$, drawn independently uniformly from [0,1]. We then assign $EQ = 1$ for years 2005-2006 to those regions with $n_r^1 \leq 0.067$. For those regions with $n_r^1 > 0.067$ and $n_r^2 \leq 0.13$ we assign $EQ = 1$ for years 2007-2009; and for regions with $n_r^1 > 0.067$, $n_r^2 > 0.13$ and $n_r^3 \leq 0.4$, $EQ = 1$ only after 2009. For the remaining regions $EQ = 0$ throughout. Because there are only 15 Chilean regions, the probability that we pick up affected regions in the data is positive, which can increase the number of times we find effects under this specification. This problem is partially mitigated by simulating the outcome variable, y_j^t. In each replication of the test and for each region and year, we assume that y_j^t is normally distributed with mean μ and variance σ^2. The parameters μ and σ^2 are, respectively, equal to the mean and variance of `Crime`, at national level and for the period considered in this work: 2005-2011.

We estimate the fixed effect model 10000 times using the same controls as in our main specification. Average results are reported in Table 9. If we consider a significance level of 1%, the percentage of rejections of the null hypothesis $\hat{\beta}_{EQ} = 0$ is 6% (that percentage increases to 13 and to 20, if we consider significance levels of 5% and 10%, respectively). Although the percentage of rejections is high, which has to do with the small number of Chilean regions (15), the average value of the estimated coefficient $\hat{\beta}_{EQ}$ is virtually equal to zero.

We can contrast this to our results where for 3 of our 6 indicators of social cohesion we reject the null-hypothesis at the 5 percent level. Given that a "random rejection" occurs with probability 0.1338 in our data, the probability that our result is generated randomly is given by

$$\binom{6}{3} 0.1338^3 * 0.8662^3 \approx 0.031.$$

Note that this assumes independence across social cohesion indicators, which seems appropriate in the absence of additional information.

Comunas. For the *comuna*-level regressions, we proceed in the same fashion. We pick 22% of Chilean *comunas* at random and impose $\texttt{EQ}=1$ on them and we artificially generate the dependent variable. We estimate the difference in difference model, with standard errors clustered at province level. In this case, we get rejections 1%, 6% and 11% of the times, depending on the critical significance level we consider. Moreover, the average value of the estimated coefficient corresponding to the interaction variable $\texttt{Post}\times\texttt{EQ}^{2010}$ is approximately equal to zero. Given that a "random rejection" occurs with probability 0.058 in our data, the probability that our result is generated randomly is given by

$$\binom{5}{3}(0.058)^3 * 0.942^2 \approx 0.0017$$

Placebo Test II We also conduct a second type of Placebo test for *comunas*. To this end we move a placebo treatment to 2013. We chose this year for two reasons. On one hand, we only have observations for the years 2009, 2011 and 2013 for our variable `Volunteering`. On the other hand, the period 2005-2009 is problematic since during the years 2005 and 2007 Chile suffered two important earthquakes: Tarapacá of magnitude Ms 7.8, and Antofagasta of Ms 7.5, respectively. Hence, the estimates could capture the effects that these earthquakes could have on the outcome variables.

We perform the difference in difference estimates assuming that `POST13` equals 1 if $t = 2013$ and zero if $t = 2011$. Then the interaction variable $\texttt{POST13}\times\ \texttt{EQ}_c^{2010}$ equals 1 if $t = 2013$ and C is a comuna affected by the Maule earthquake in 2010 as before. The estimated coefficients reported in Table 10 show that the "placebo interaction" $\texttt{POST13}\times\ \texttt{EQ}_c^{2010}$ is insignificant throughout.

Individual-level Regressions Finally, Table 11 shows individual level regressions based on repeated cross-sections from the CASEN waves 2009, 2011 and 2013 as well as waves 2008, 2009 and 2012 of the Citizen Safety Survey.

The regressions in Columns (1) and (2) replicate our diff-in-diff approach at the *comuna* level. Column (1) reports estimates based on the entire sample of 520787 respondents, while column (2) excludes (in each wave) all individuals that have moved between *comunas* in the last five years. The comparison of both regressions discards the possibility that the detected effects are driven by migration of the populatoin across regions.

Columns (3) and (4) exploit data from the Citizen Safety Survey. In particular we use question (P22_1_1 in 2008 and 2009 and P20_1_1 in 2012): "Have you, or any member of your family, been victim of a crime?". The possible answers are (1) Yes, (2) No, (3) Do not Know and (4) No Answer. The variable $\texttt{Victim of Crime}_{i,t}$ equals one if individual i, in period t, choose option (1). Since the crime could happen in locations different than the victims' residence, we also use question (P114_1_1 in 2008 and 2009 and P54_1_1 in 2012): "Have you, or any member of your family, been victim of a burglary?". Our variable, $\texttt{Burglary}_{i,t}$ equals one if individual i, in period t, answers "Yes". Related to this question, individuals were asked where this crime took place. The possible answers are (1) At their home or neighborhood, (2) At their *comuna*, (3) In another *comuna*, (4) In another region of the Country, (5) In another Country, (88) Do not know, (99) No answer. We then run individual level probit regressions based on repeated cross sections from the waves 2008, 2009 and 2012. These regressions replicate the diff-in-diff approach at the *comuna* level. Column (3) uses $\texttt{Victim of Crime}_{i,t}$ as dependent variable, while Column (4) uses $\texttt{Burglary}\ _{i,t}$ as dependent variable. We exclude (in each wave) those cases in which the location of the crime was different that the victim's home, neighborhood or *comuna*.

References :

[S1] Friedkin, N. (2004), "Social cohesion", *Annual Review of Sociology* 30, 409-435.
[S2] Friedman, J. and A. Hofman (2013), "Inequality and the Top of the Income Distribution in Chile 1990-2012: Questioning the Consensus", SSRN working paper 2242259.
[S3] OECD (2012), "Social cohesion indicators" in: *Society at a Glance: Asia/Pacific 2011*, OECD Publishing.
[S4] US Geological Survey, "The severity of an earthquake" (http://pubs.usgs.gov/gip/earthq4/severitygip.html)

	Unit of observation: Chilean regions					
	Charity		Volunteering		Voting	
	(1)	(2)	(3)	(4)	(5)	(6)
**Variable**						
EQ	0.34**		0.154		0.419*	
	(0.132)		(0.243)		(0.232)	
DISTEQ		-0.128**		-0.599***		-0.185
		(0.058)		(0.172)		(0.226)
Lag Gini	-0.070	0.011	1.461***	-0.033	0.323**	0.049
	(0.047)	(0.094)	(0.243)	(0.350)	(0.124)	(0.515)
Lag Log Income	-0.008	0.132	-3.178***	0.117	-1.867***	-0.011
	(0.152)	(0.154)	(0.714)	(0.559)	(0.521)	(0.669)
Lag Log poverty	0.169	0.034	0.928	0.822	0.084	0.007
	(0.184)	(0.182)	(0.588)	(1.001)	(0.286)	(0.833)
Share women	-0.007	-0.007	-0.187	-0.061	-0.305	-0.105
	(0.143)	(0.085)	(0.143)	(0.226)	(0.191)	(0.237)
Lag Migration Rate	0.363	0.669*	1.246	2.248	1.437*	1.147*
	(0.265)	(0.313)	(1.699)	(1.361)	(0.676)	(0.651)
Constant	0.068	0.116***	-0.765***	-0.059	0.314	0.629
	(0.045)	(0.017)	(0.222)	(0.105)	(0.192)	(0.415)
Observations	56	71	28	30	28	28
Regions	15	15	15	15	15	15
R-squared	0.481	0.479	0.911	0.700	0.934	0.837
Region Fixed Effects	YES	YES	YES	YES	YES	YES
Year Dummies	07,10	07,10	11	11	12	12

Table 3: Fixed effect estimates: values shown are the estimated standardized coefficients (standard errors in parenthesis) of the earthquake-related variable from region regressions; both the dependent and control variables have been converted to z-scores. Controls include lagged (three periods back) Gini coefficient, migration rate, income and poverty. Significance level (***) 1%, (**) 5% and (*) 10%.

	Unit of observation: Chilean regions					
	Crime		Corruption		Suicides	
	(7)	(8)	(9)	(10)	(11)	(12)
**Variable**						
EQ	0.452**		-0.099		-0.258	
	(0.189)		(0.175)		(0.328)	
DISTEQ		0.146**		0.009		0.261***
		(0.063)		(0.072)		(0.084)
Lag Gini	-0.220	-0.466***	0.150	0.098**	-0.106	0.073
	(0.130)	(0.093)	(0.098)	(0.037)	(0.267)	(0.198)
Lag Log Income	0.483*	0.929***	-0.526*	-0.024**	-0.352	-0.383
	(0.229)	(0.188)	(0.285)	(0.110)	(0.442)	(0.348)
Lag Log poverty	0.825**	0.544**	0.467**	-0.047	-0.071	0.751*
	(0.313)	(0.235)	(0.214)	(0.162)	(0.945)	(0.413)
Share women	0.095	0.200	-0.173	-0.017	0.035	0.138
	(0.160)	(0.139)	(0.112)	(0.041)	(0.339)	(0.199)
Lag Migration Rate	0.268	1.055**	-0.710	-0.064	-0.620	-2.129**
	(1.149)	(0.401)	(0.407)	(0.342)	(2.200)	(0.983)
Constant	0.841***	0.424***	-0.158*	-0.147***	0.129	0.116
	(0.093)	(0.054)	(0.082)	(0.018)	(0.173)	(0.083)
Observations	54	84	69	99	54	84
Regions	15	15	15	15	15	15
R-squared	0.544	0.658	0.139	0.091	0.165	0.158
Region Fixed Effects	YES	YES	YES	YES	YES	YES
Year dummies	05,07,10	05,07,10	05,07,10	05,07,10	05,07,10	05,07,10

Table 4: Fixed effect estimates: values shown are the estimated standardized coefficients (standard errors in parenthesis) of the earthquake-related variable from region regressions; both the dependent and control variables have been converted to z-scores. Controls include lagged (three periods back) Gini coefficient, migration rate, income and poverty. Significance level (***) 1%, (**) 5% and (*) 10%.

	Unit of observation: Chilean regions					
	Charity		Volunteering		Voting	
	(1)	(2)	(1)	(2)	(1)	(2)
**Variable**						
EQ	0.132**		0.023***		0.003	
	(0.052)		(0.006)		(0.015)	
DISTEQ		-0.001		-0.002***		0.0004
		(0.001)		(0.001)		(0.001)
Lag Comp. 1	0.041	-0.072	-0.088***	0.005	-0.053*	-0.021
	(0.049)	(0.049)	(0.006)	(0.014)	(0.028)	(0.024)
Lag Comp. 2	0.002	0.019	0.034***	0.011	0.001	-0.006
	(0.029)	(0.027)	(0.003)	(0.019)	(0.005)	(0.025)
Lag Comp. 3	-0.013	-0.013***	-0.001	0.026	0.047**	-0.021
	(0.036)	(0.033)	(0.012)	(0.027)	(0.017)	(0.060)
Constant	6.541***	6.552***	0.337***	0.395***	0.342***	0.426***
	(0.026)	(0.043)	(0.007)	(0.015)	(0.032)	(0.029)
Observations	56	71	28	30	28	28
Regions	15	15	15	15	15	15
R-squared	0.310	0.498	0.942	0.648	0.895	0.821
Year dummies	07, 10	07, 10	11	11	12	12

Table 5: Principal Component Analysis estimates. Models (1) and (2) corresponds to exposure to earthquake and distance in years since the last earthquake, respectively. Both models include lagged values of the components. Significance level (***) 1%, (**) 5% and (*) 10%.

	Unit of observation: Chilean regions					
	Crime		Suicides		Corruption	
	(1)	(2)	(1)	(2)	(1)	(2)
**Variable**						
EQ	−0.069***		-0.198		-0.00004	
	(0.023)		(0.536)		(0.001)	
DISTEQ		0.003***		0.045*		-0.00001
		(0.001)		(0.024)		(0.00005)
Lag Comp. 1	0.097	0.047	-1.177	-2.267*	-0.003	-0.021
	(0.076)	(0.035)	(2.043)	(1.127)	(0.002)	(0.024)
Lag Comp. 2	-0.025	0.018	-1.058	0.058	-0.002***	-0.001
	(0.041)	(0.035)	(1.009)	(0.464)	(0.001)	(0.001)
Lag Comp. 3	-0.121**	-0.084	0.481	-1.339	-0.002	0.0002
	(0.041)	(0.048)	(1.911)	(0.948)	(0.001)	(0.001)
Constant	8.244***	8.052***	11.732***	11.764***	0.003**	0.005***
	(0.023)	(0.017)	(0.617)	(0.596)	(0.001)	(0.001)
Observations	54	84	54	84	69	99
Regions	15	15	15	15	15	15
R-squared	0.418	0.497	0.274	0.109	0.168	0.080
Year dummies	05,07,10	05,07,10	05,07,10	05,07,10	05,07,10	05,07,10

Table 6: Principal Component Analysis estimates. Models (1) and (2) corresponds to exposure to earthquake and distance in years since the last earthquake, respectively. Both models include lagged values of the componentes. Significance level (***) 1%, (**) 5% and (*) 10%.

	Unit of observation: Chilean *comunas*									
	Life Sat.		Trust		Volunteering		Crime		Suicides	
POST	−0.813***		−0.157		0.465***		0.186*		0.111	
	(0.245)		(0.221)		(0.101)		(0.109)		(0.0963)	
EQ_c^{2010}	-0.333		-0.277		-0.404**		0.134		-0.146*	
	(0.251)		(0.272)		(0.155)		(0.0982)		(0.0752)	
$POST \times EQ_c^{2010}$	0.742***		0.110		0.265**		-0.162**		0.0479	
	(0.267)		(0.183)		(0.128)		(0.0620)		(0.111)	
DISTEQ		−0.006		-0.003		-0.0625**		0.0321**		-0.0105
		(0.006)		(0.006)		(0.0314)		(0.0143)		(0.0394)
Lag Gini Coefficient	0.278**	−0.199*	-0.099	-0.011	-0.0380	-0.406***	-0.0715	0.0230	-0.0204	-0.0687
	(0.105)	(0.105)	(0.178)	(0.132)	(0.0729)	(0.136)	(0.0698)	(0.0390)	(0.0359)	(0.0863)
Lag Log Income	0.137	0.056	0.066	-0.076	0.168	0.901***	0.210	0.0143	0.125	0.212
	(0.129)	(0.120)	(0.187)	(0.160)	(0.111)	(0.302)	(0.192)	(0.0913)	(0.0767)	(0.213)
Lag Poverty Index	0.049	0.045	-0.071	−0.279**	0.320***	0.497***	0.111	-0.0421	0.173***	0.117
	(0.166)	(0.157)	(0.170)	(0.136)	(0.0931)	(0.152)	(0.108)	(0.0496)	(0.0495)	(0.128)
Share Women	-0.097	-0.075	-0.025	0.057	-0.0410	-0.00785	-0.00952	-0.0204	-0.0519	-0.106**
	(0.087)	(0.078)	(0.074)	(0.088)	(0.0338)	(0.0366)	(0.0222)	(0.0166)	(0.0405)	(0.0467)
Lag Migration Rate	-0.072	−0.029	0.028	0.060*	-0.104***	-0.322	-0.0595	0.00837	-0.106***	0.188
	(0.055)	(0.039)	(0.065)	(0.032)	(0.0339)	(0.218)	(0.0636)	(0.0581)	(0.0347)	(0.117)
Years Schooling					-0.450***	-0.0499	0.548***	0.0333	-0.175***	-0.178
					(0.111)	(0.0806)	(0.0909)	(0.0365)	(0.0549)	(0.126)
Constant	0.289	0.188	0.326*	-0.144	-0.140	-0.00276	-0.0537	0.276***	-0.0701	0.00465
	(0.183)	(0.245)	(0.185)	(0.221)	(0.0858)	(0.0755)	(0.0636)	(0.0293)	(0.0685)	(0.0618)
Observations	227	282	227	282	640	640	960	960	960	960
R-squared	0.205	0.123	0.044	0.07	0.350	0.280	0.484	0.376	0.076	0.033
Diff in Diff	YES	NO	YES	NO	YES	NO	YES	NO	YES	NO
Fixed Effects	NO	YES	NO	YES	NO	YES	NO	YES	NO	YES
Comunas	98	105	98	105	320	320	320	320	320	320
Year dummies	08-11	06-11	08-11	06-11	-	09	09	06,09	09	06,09

Table 7: Values shown are the estimated standardized coefficients (standard errors in parenthesis) of the earthquake-related variable from *comuna* level regressions; both the dependent and control variables have been converted to z-scores. Life Satisfaction and Trust include additional controls from the Latinobarómetro database (age, political position, education category and income constraint). Significance level (***) 1%, (**) 5% and (*) 10%.

	Unit of observation: Chilean *comunas*									
	`Life Sat.`		`Trust`		`Volunteering`		`Crime`		`Suicides`	
	(1)	(2)	(1)	(2)	(1)	(2)	(1)	(2)	(1)	(2)
**Variable**										
`POST`	−0.944***		-0.164		0.089***		−0.309		5.122**	
	(0.307)		(0.312)		(0.017)		(0.234)		(2.380)	
$\mathtt{EQ}_c^{2010}$	−0.042***		-0.271		-0.267		0.134**		−1.874**	
	(0.233)		(0.276)		(0.014)		(0.057)		(0.890)	
$\mathtt{POST} \times \mathtt{EQ}_c^{2010}$	0.778**		0.090		0.025**		−0.106***		0.652	
	(0.293)		(0.175)		(0.012)		(0.039)		(1.302)	
$Dist_EQ$		−0.011**		-0.001		−0.001**		0.002**		−0.018
		(0.005)		(0.006)		(0.0003)		(0.001)		(0.047)
**Controls**										
Lag Comp. 1	-0.008	-0.049	0.062	0.056	−0.04***	-0.017	0.232***	0.026	−2.032***	-0.355
s.e.	(0.053)	(0.052)	(0.051)	(0.037)	(0.004)	(0.013)	(0.021)	(0.024)	(0.374)	(1.116)
Lag Comp. 2	−0.079*	-0.052	0.021	0.067	0.023*	−0.048*	−0.125	0.027	1.016	3.746**
s.e.	(0.045)	(0.050)	(0.062)	(0.054)	(0.012)	(0.029)	(0.084)	(0.039)	(0.726)	(1.817)
Lag Comp. 3	−0.141**	−0.142**	-0.024	-0.017	0.001	0.007	0.094***	−0.026*	0.343	−1.738**
s.e.	(0.059)	(0.056)	(0.062)	(0.049)	(0.006)	(0.007)	(0.027)	(0.014)	(0.487)	(0.827)
Lag Comp. 4	−0.142**	−0.097*	-0.019	-0.053	−0.003	−0.017**	−0.055	-0.002	−0.569	2.055*
s.e.	(0.057)	(0.053)	(0.038)	(0.037)	(0.003)	0.009	(0.050)	(0.027)	(0.362)	(1.170)
Lag Comp. 5	0.263*	0.175	-0.002	0.020						
s.e.	(0.131)	(0.115)	(0.149)	(0.132)						
Lag Comp. 6	0.023	-0.015	-0.066	-0.100						
s.e.	(0.059)	(0.047)	(0.095)	(0.067)						
Constant	0.472**	0.469	0.336*	0.160	0.364***	0.388***	7.857***	7.789***	11.072***	19.115***
s.e.	(0.177)	(0.322)	(0.190)	(0.303)	(0.008)	(0.010)	(0.167)	(0.050)	(1.499)	(2.354)
Observations	227	282	227	282	640	640	960	960	960	960
Comunas	98	105	98	105	320	320	320	320	320	320
R-squared	0.10	0.08	0.037	0.04	0.350	0.250	0.404	0.370	0.060	0.020
Year dummies	08-11	06-11	08-11	06-11	06, 09	06, 09	06,09	06,09	06,09	06,09

Table 8: Principal Component Analysis. Models (1) Diff in Diff estimates and Model (2) Fixed Effect estimates (independent variable distance in years since the last earthquake). Both models include lagged values of the componentes. Significance level *** 1%, ** 5% and * 10%.

	Regions		*Comunas*	
Confidence level	Average $\hat{\beta}_{EQ}$	% of rejections	Average $\hat{\beta}_{EQ}$	% of rejections
1%	0.0013	5.94	0.0018	1.07
5%	0.0013	13.38	0.0018	5.8
10%	0.0013	19.55	0.0018	10.52
5%	Pr(Result randomly generated) ≈ 0.031		≈ 0.0017.	

Table 9: Placebo test I: Random Assignment of `EQ`= 1 on Regions/Comunas.

	Units of observations Chilean Comunas		
	**Life Sat**	**Volunteering**	**Crime**
**Variables**			
POST13	-1.835^{***}	0.0202^{**}	0.673^{***}
	(0.181)	(0.00768)	(0.0894)
EQ^{2010}	0.048	-0.0184	-0.0300
	(0.209)	(0.0153)	(0.0827)
POST13 $\times$ EQ^{2010}	-0.232	-0.0018	0.0549
	(0.179)	(0.0104)	(0.112)
**Controls**			
Lag Gini coefficient	-4.479^{**}	-0.0176	-0.844^{**}
	(1.696)	(0.079)	(0.381)
Lag (log) Income	0.806^{*}	0.0224	0.284^{*}
	(0.447)	(0.0275)	(0.146)
Lag Poverty	2.821	0.463^{***}	0.491
	(2.502)	(0.115)	(0.558)
Women	0.223	-0.0789	-0.578
	(0.438)	(0.116)	(0.380)
Lag Net Migr. Rate	-0.2467	-0.0416^{**}	-0.0367
	(0.298)	(0.0157)	(0.0929)
Years Schooling		-0.360^{***}	1.004^{***}
		(0.0846)	(0.179)
Constant	-8.623	0.932^{***}	2.731^{**}
	(5.392)	(0.196)	(1.330)
Observations	95	640	640
Comunas	56	320	320
R-squared	0.852	0.340	0.630

Table 10: Placebo Test II. The variable POST13 equals 1 if $t = 2013$ and zero if $t = 2011$, $EQ^{2010} = 1$ if Comuna C was hit by the 8.8-magnitude earthquake in 2010 and POST13 $\times$ EQ^{2010} is the interaction between POST13 and EQ^{2010}. Life Satisfaction includes additional controls from the Latinobarómetro database (age, political position, education category and income constraint). Significance level $(***)$ 1%, $(**)$ 5% and $(*)$ 1%.

	Volunteering		Crime	
	(1)	(2)	(3)	(4)
	Full Sample	Non-Migrants	Victim of Crime	Burglary
POST	0.0853***	0.0858***	-0.267***	-0.0545
	(0.0321)	(0.0328)	(0.0227)	(0.0482)
EQ^{2010}	0.0932**	0.0797*	0.000293	0.230***
	(0.0460)	(0.0474)	(0.0407)	(0.0481)
$POST \times EQ^{2010}$	0.105**	0.115***	-0.143***	-0.186**
	(0.0437)	(0.0438)	(0.0428)	(0.0784)
Gender	0.0560***	0.0627***	-0.00590	-0.109***
	(0.00931)	(0.00986)	(0.0165)	(0.0294)
Educ. Mid Level	-0.0851***	-0.0856***	0.144***	0.0923***
	(0.0168)	(0.0186)	(0.0270)	(0.0318)
Educ. University	-0.134***	-0.142***	0.232***	0.113***
	(0.0308)	(0.0323)	(0.0276)	(0.0311)
Years Schooling	0.00784***	0.00828***		
	(0.00243)	(0.00256)		
Urban	0.375***	0.377***		
	(0.0291)	(0.0298)		
Age	0.00832***	0.00825***	-0.00857***	-0.00524***
	(0.000337)	(0.000343)	(0.000621)	(0.000933)
Income	-2.18e-08	-7.29e-09		
	(1.44e-08)	(1.32e-08)		
Constant	-1.735***	-1.731***	-0.104***	-1.360***
	(0.0619)	(0.0634)	(0.0403)	(0.0705)
Observations	520787	482487	77688	76072
Dummy 2013	YES	YES	-	-
Quintile Dummies	-	-	YES	YES

Table 11: Individual level probit estimations using repeated cross sections (CASEN waves 2009, 2011 and 2013) of `Volunteering` in Columns (1) and (2) and data from waves 2008, 2009 and 2012 of the Citizen Safety Survey in Columns (3) and (4). Column (1) considers all individuals; Column (2) considers a subsample of individuals who have been living in the same *comuna* during the last 5 years. In Column (3) the endogenous variable measures whether the individual was victim of a crime and in column (4) whether they were victim of a burglary. Standard errors (in parentheses) clustered at comuna level. Significance (***) $p < 0.01$, (**) $p < 0.05$, (*) $p < 0.1$
